# Effects of strobe light stimulation on postnatal developing rat retina

**DOI:** 10.1007/s00221-013-3786-8

**Published:** 2013-11-30

**Authors:** Jung-a Shin, Eojin Jeong, In-Beom Kim, Hwa-young Lee

**Affiliations:** 1 Department of Anatomy, School of Medicine, Ewha Womans University, 911-1 Mok5dong, Yang-chon, Seoul, 158-710 Korea; 2 Department of Anatomy, College of Medicine, The Catholic University of Korea, Seoul, 137-701 Korea

**Keywords:** Development, Dopaminergic amacrine cell, Retina, Strobe light, Tyrosine hydroxylase

## Abstract

The nature and intensity of visual stimuli have changed in recent years because of television and other dynamic light sources. Although light stimuli accompanied by contrast and strength changes are thought to have an influence on visual system development, little information is available on the effects of dynamic light stimuli such as a strobe light on visual system development. Thus, this study was designed to evaluate changes caused by dynamic light stimuli during retinal development. This study used 80 Sprague-Dawley rats. From eye opening (postnatal day 14), half of the rats were maintained on a daily 12-h light/dark cycle (control group) and the remaining animals were raised under a 12-h strobe light (2 Hz)/dark cycle (strobe light-reared group). Morphological analyses and electroretinogram (ERG) were performed at postnatal weeks 3, 4, 6, 8, and 10. Among retinal neurons, tyrosine hydroxylase-immunoreactive (TH-IR, dopaminergic amacrine cells) cells showed marked plastic changes, such as variations in numbers and soma sizes. In whole-mount preparations at 6, 8, and 10 weeks, type I TH-IR cells showed a decreased number and larger somata, while type II TH-IR cells showed an increased number in strobe-reared animals. Functional assessment by scotopic ERG showed that a-wave and b-wave amplitudes increased at 6 and 8 weeks in strobe-reared animals. These results show that exposure to a strobe light during development causes changes in TH-IR cell number and morphology, leading to a disturbance in normal visual functions.

## Introduction

Changes in the visual environment affect visual system development. Dark rearing is thought to decrease the intensity of immunohistochemical staining for dopaminergic cells (Kato et al. 1980), reduce storage of dopamine (Parkinson and Rando 1983), and block both maturational loss of ON–OFF responsive retinal ganglion cells and the pruning of their dendrites (Tian and Copenhagen 2003). Abnormal horizontal cell processes were found in mice reared in a no-contrast environment (Lee et al. 2008). Binocular deprivation of pattern vision impairs maturation of the α-retinal ganglion cell (Burnat et al. 2012). Furthermore, chronic exposure to a bright luminous environment induces retinopathy in juvenile rats (Joly et al. 2006).

The use of information technology has become very common, resulting in the continuous exposure of individuals to various visual stimuli. Although the human retina has adult-like cytoarchitecture at birth (Hollenberg and Spira 1972, 1973; Spira and Hollenberg 1973), the maturation of retinal function occurs over an extended period of time (Hooks and Chen 2007). Excessive exposure to visual stimuli could have adverse effects during the developmental period. American preschool children spend more than 31 h per week watching television and more than 5 h per week exposed to other media such as videogames and computers (Lee et al. 2009). These visual stimuli occasionally may result in unexpected events such as myopia, asthenopia, exophoria, and convergence insufficiency (Gratton et al. 1990; Murata et al. 1991; Basso et al. 2006; Takada et al. 1999).

Stroboscopic illumination has various side effects. Illumination in both eyes disrupted binocular map development in the optic tectum of the *Xenopus* frog (Brickley et al. 1998) and prevented sharpening of the retinotectal projection in goldfish (Schmidt and Buzzard 1993). The number of directionally selective responses in the superficial superior colliculus decreased in the strobe-reared guinea pig (Thornton et al. 1996). Strobe rearing prevented the normal development of the vestibulo-ocular reflex in the chicken (Goode et al. 2001), and strobe-reared cats were found to be more myopic than normal cats (Cremieux et al. 1989). Similarly, the function of the brain and the refractive error of the eye have been studied, but little information is available on changes in the retina induced by strobe light. Our previous study (Shin et al. 2012) showed that rearing guinea pigs under a strobe light induced thinning of the outer nuclear layer (ONL) and abnormal axon processes in rod bipolar cells.

The purpose of this study was to test whether rearing animals in stroboscopic illumination yield plastic change in the developing rat retina. To this end, we studied morphology and retinal function after rearing rats in stroboscopic illumination.

## Materials and methods

### Animals

This study used 80 Sprague-Dawley (SD) rats: 40 rats for the control group and 40 rats for the strobe-reared model. The animals were divided into control and experimental exposure groups after birth, and reared in cages (60 cm × 40 cm × 40 cm) with 30-W incandescent lamp located at the top of each cage as an illumination sources from eye opening (postnatal [P]day 14). Strobe-reared groups were illuminated stroboscopically at rate of 2 Hz and an intensity of 150 Lux for 12 h/day. The control animals were reared under a 12-h light/dark cycle with the same general living and exposure conditions (duration, location, and light intensity). Rats were killed at the following ages: P 3 weeks (*n* = 16), 4 weeks (*n* = 16), 6 weeks (*n* = 16), 8 weeks (*n* = 16), and 10 weeks (*n* = 16). All animals were treated in accordance with the Institutional Animal Care and Use Committee of Ewha Womans University School of Medicine.

### Tissue preparation

Animals were deeply anaesthetized by intraperitoneal injection of zolazepam (12.5 mg/kg body weight), and the eyes were enucleated. Animals were killed with an overdose of zolazepam. The anterior segments of the eyes were removed, and the eyecups were fixed by immersion in 4 % paraformaldehyde in 0.1 M phosphate saline buffer (PBS, pH 7.4) for 2 h. Following fixation, the retinas were carefully dissected and were transferred to 30 % sucrose for 24 h at 4 °C. Radial cuts were made to facilitate flattening. The samples were frozen in liquid nitrogen, thawed, and rinsed in 0.1 M PBS.

### Morphological study

At each of the time points described above, two superior temporal retinas were dehydrated and embedded in Epoxy resin. The 1-μm-thick sections were cut by an ultramicrotome using a diamond knife. Retinal sections were stained with toluidine blue and were mounted on slides, and the images were obtained using an Olympus microscope.

### Immunohistochemistry

For fluorescence immunohistochemistry, 40-μm-thick vibratome sections and whole-mount preparations were blocked in 10 % normal goat serum in PBS for 1 h at room temperature (25 °C). Subsequently, the sections were incubated with primary and secondary antibody dilutions prepared in 0.1 M PBS with 0.5 % Triton X 100. The sections were incubated with a rabbit polyclonal antibody directed against tyrosine hydroxylase (TH) (Chemicon International, Temecula, CA.; dilution 1:1,000) for 1–3 days at 4 °C and washed in PBS for 45 min (3 × 15 min). Afterward, the retinas were incubated for 2 h in Cy3-conjugated goat anti-rabbit IgG (Jackson Immuno Research, West Grove, PA; dilution 1:2,000). The retinal tissues were thoroughly washed with PBS and were mounted with VECTASHIELD with DAPI H-1200 (Vector Laboratories).

Fluorescent images were acquired using a Zeiss LSM 510 confocal microscope (Carl Zeiss Co. Ltd., Oberkohen, Germany). Images were converted into JPEG format and were processed for contrast level adjustment using Adobe Photoshop v. 7.0 (Adobe Systems, San Jose, CA).

### Analysis of TH-IR cell morphology

**Fig. 1:**
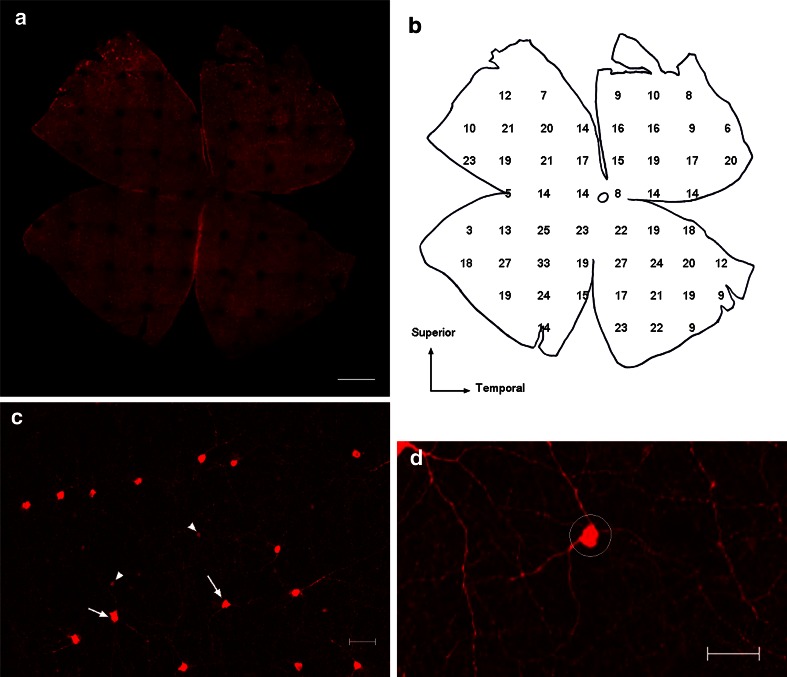
Images of whole-mount preparations processed for TH immunoreactivity in the left control retina (**a**, **c**, **d**) and map of the image shown in *panel a* (**b**).The *large circle* represents the position of the optic disk, and each *number* represents the number of type I TH-IR cells counted in an area of 1 mm^2^ in **b**. The *arrows* indicate type I TH-IR cells, and the *arrowheads* indicate type II TH-IR cells in **c**. The diameter of the dendrite was measured at a distance of 20 μm from the center of the cell body (**d**) (TH, tyrosine hydroxylase; TH-IR, tyrosine hydroxylase immunoreactive. *Scale bar* = 1 mm in **a,** and 50 μm in **c**, **d**)

The numbers of tyrosine hydroxylase-immunoreactive (TH-IR) neuronal populations were counted in whole-mount preparations of control and experimental retinas. The left retinas (*n* = 3) from each group at the specific experimental time point were used for analysis. Photographs (1 × 1 mm, *z*-axis = 65 μm) were taken under a 10 × objective lens and stacked to ensure that all of the TH-IR cells were visible. The representative image of a sample is shown in Fig. 1a.

The TH-IR cells in the rat retina are composed of 2 major classes (Nguyen-Legros et al. 1983, 1984; Versaux-Botteri et al. 1986): Type I TH-IR cells are usually labeled heavily and have relatively large somata, whereas type II TH-IR cells have smaller somata and are usually labeled lightly (Fig. 1c). Because the soma size differs among the retinal area, the numbers of type I and type II TH-IR neurons were manually measured by 2 separate persons instead of by image analysis software. The numbers in Fig. 1b represent cell counts/mm^2^. The vertical sum of the numbers is represented on the *y*-axis of the graphs in Fig. 4a, and the total sum of the numbers is represented in Fig. 4b, c.

The soma area and diameter of the dendrites were measured in the central part of the superior temporal retina, and 50 cells were measured for each time point. The soma area was measured with free shape drawing mode in Zeiss LSM image examiner v. 4.0. The dendrite diameter was measured at a distance of 20 μm from the center of the cell body (Fig. 1d).

Statistical analysis of TH-IR cell number was performed using the Mann–Whitney *U* test with the Statistical Package for Social Sciences (SPSS version 16.0). *p* values < 0.05 were considered significant.

### Electroretinogram

Five rats were used at each time point for electroretinogram (ERG) experiments. All rats were dark adapted for 12 h before ERG experiments and were prepared for recording under dim red light (*λ* > 600 nm). Animals were anaesthetized by intraperitoneal injection of zolazepam (12.5 mg/kg body weight) and were lightly secured to a stage to ensure a stable position for ERG recording. The top of the stage was fixed to the position where the animal’s eye faced the flashing light at a 20-cm distance. The depth of anesthesia was sensitively monitored online by inspection of the ERG signal baseline. The cornea was protected with hydroxypropyl methylcellulose gel before a gold ring contact electrode was placed on it. A reference electrode and a ground electrode were placed subcutaneously in the ear and in the tail, respectively. The stimuli were brief white flashes (xenon arc discharge) delivered via a Ganzfeld integrating sphere (Model UTAS-3000, LKC Technologies, Gaithersburg, MD, USA). Stimulus intensities were measured using a calibrated photometer with a scotopic luminosity filter in place. Scotopic ERGs obtained for all intensities above 0.9 log (cd s) m^−2^ were recorded as single flash responses (Gao et al. 2010; Chen et al. 2009). Each record was an average of 3 responses obtained with a 2-s interstimulus interval. Signals were recorded with band-pass filters of 1–3 Hz. The amplitude of the a-wave was measured from the baseline to the a-wave trough, and the b-wave was measured from the maximum a-wave trough to the maximum b-wave peak.

## Results

### Histology in strobe-reared retinas

**Fig. 2:**
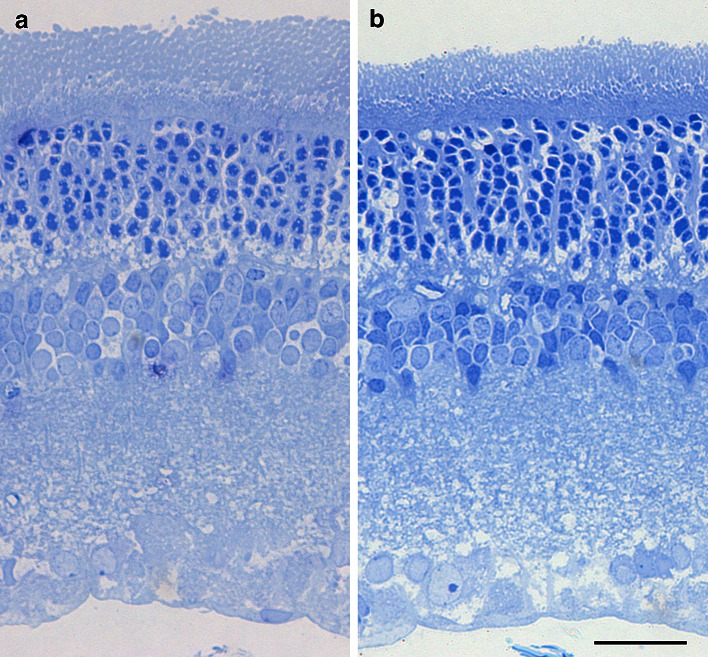
Images from 1-μm-thick, vertical semi-thin sections stained with toluidine blue in control (**a**) and strobe-reared retinas (**b**) at postnatal 6 weeks. No differences in retinal thickness and other gross morphology were apparent (*PR* photoreceptor,* ONL* outer nuclear layer,* OPL* outer plexiform layer,* INL* inner nuclear layer,* IPL* inner plexiform layer,* GCL* ganglion cell layer.* Scale bar *= 20 μm)

To determine whether the strobe-reared condition affected the morphology of the retina, we conducted toluidine blue staining on vertical sections at P 6 weeks. No differences were observed in retinal thickness and other gross morphology (Fig. 2a, b).

### Changes in TH-IR cell morphology

**Fig. 3:**
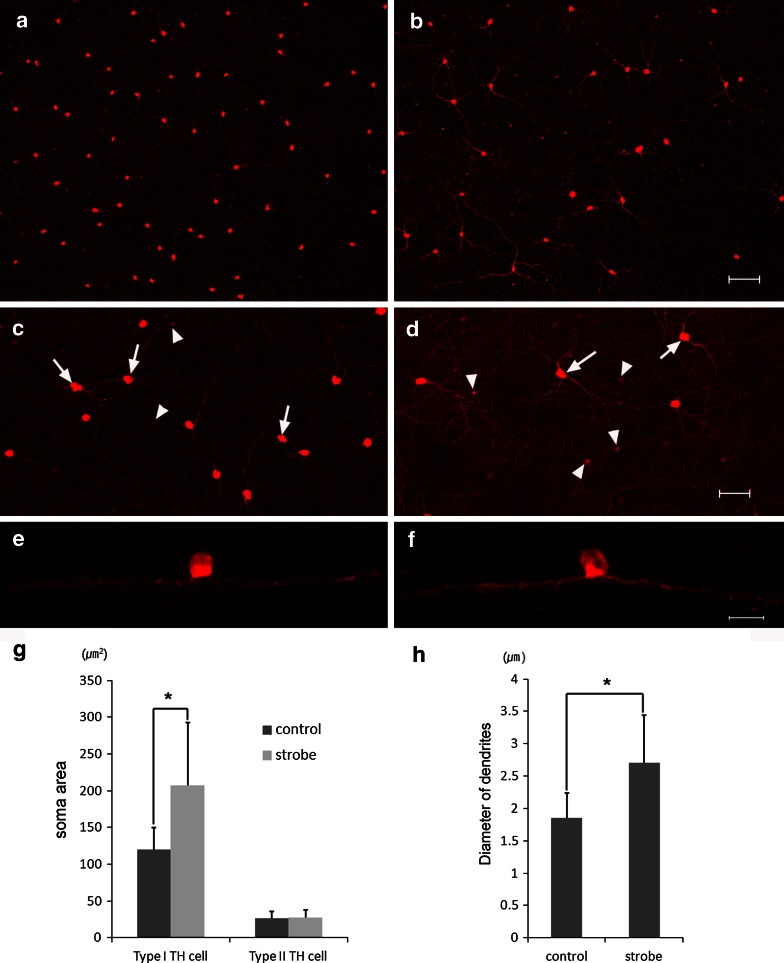
Images of whole-mount preparations (**a**–**d**) and 40-μm-thick vertical sections (**e**, **f**) processed for TH immunoreactivity in the superior temporal region of the control (**a**, **c**, **e**) and the strobe-reared retina (**b**, **d**, **f**) at postnatal 6 weeks. Histograms of the soma area for type I TH-IR cells and type II TH-IR cells (**g**) and histogram of the diameter of dendrites for type I TH-IR cells (**h**) in the superior temporal retina. The *arrows* indicate type I TH-IR cells, and the *arrowheads* indicate type II TH-IR cells in **c**, **d**. The number of type I TH-IR cells decreased, and the number of type II TH-IR cells increased in the strobe-reared retina (**b**, **d**). No differences were apparent in the vertical section (**e**, **f**). In type I TH-IR cells, the soma area (**g**) and the diameter of dendrites (**h**) increased significantly in the strobe-reared retina. No significant differences were observed in type II TH-IR cells (**g**) (TH, tyrosine hydroxylase; TH-IR, tyrosine hydroxylase immunoreactive.* Scale bar* = 100 μm in **a**, **b**, 50 μm in **c**, **d,** and 20 μm in **e**, **f**) (*p < 0.05)

One of the most prominent changes occurred in type I TH-IR cells. In the whole-mount preparation, the soma size increased and the number decreased in the strobe-reared retina at P 6 weeks (Fig. 3a, b). The soma size of type I TH-IR cells increased approximately 73.0 % in the strobe-reared rats compared to the control rats (119.75 [29.82] μm^2^ in the control retina and 207.17 [85.82] μm^2^ in the strobe-reared retina) in the superior temporal region of the whole-mount preparations (Fig. 3e, g). Furthermore, type I TH-IR cells showed stouter dendrites. The diameter of the dendrites increased about 46.5 % in the strobe-reared rats compared to the control retina (1.85 [0.37] μm in the control retina and 2.71 [0.73] μm in the strobe-reared retina) (Fig. 3f). Significant differences were not observed in the vertical sections (Fig. 3c, d). No significant difference was observed in the soma size of type II TH-IR cells (Fig. 3e).

### Changes in TH-IR cell number

**Fig. 4:**
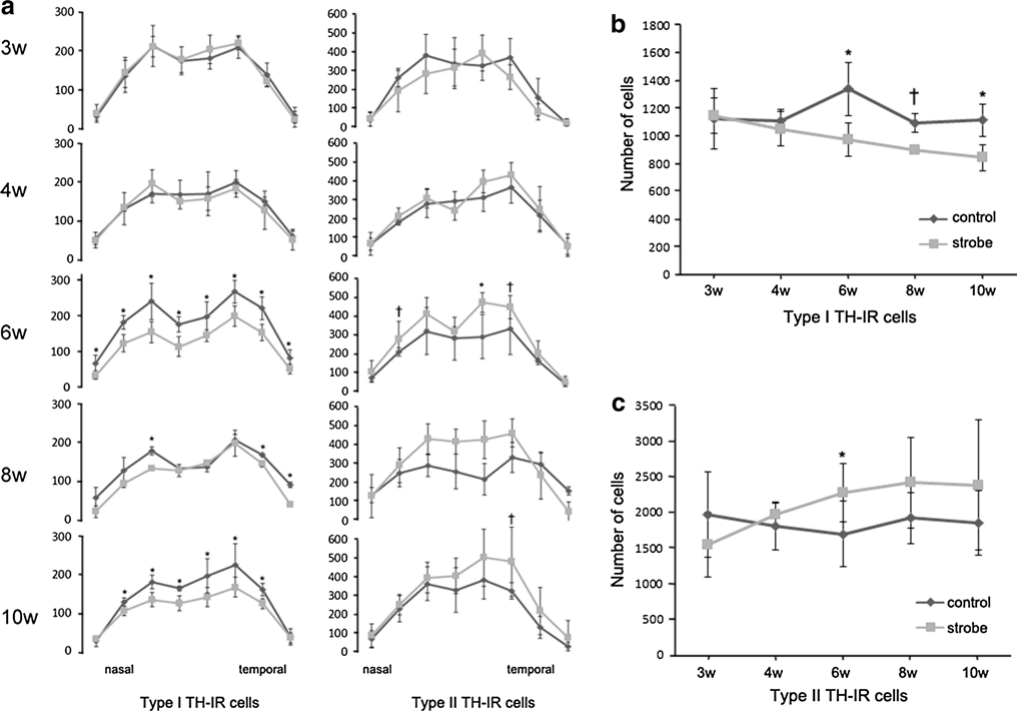
Histograms of type I and type II TH-IR cell numbers by location (**a**). Histogram of total type I TH-IR cell numbers (**b**) and total type II TH-IR cell numbers (**c**) in the whole-mount retina. The strobe-reared retina showed a decrease in type I cell number and an increase in type II cell number at postnatal 6, 8, and 10 weeks (**a**). The total cell number of the strobe-reared retina tended to show a decrease in type I cells and an increase in type II cells according to time point (**b**, **c**) (TH-IR, tyrosine hydroxylase immunoreactivity) (*p < 0.05, ^†^ *p* < 0.1)

To clarify the time course of the changes in TH-IR cells, we counted the number of TH-IR cells at P 3, 4, 6, 8, and 10 weeks. At P 6 weeks, the number of type I TH-IR cells was significantly decreased in most regions of strobe-reared retinas compared to control retinas. The number of type II TH-IR cells increased, but only a few regions showed statistical differences (Fig. 4a). Similar changes in TH-IR cells were found at P 8 and 10 weeks, but no significant differences were observed at P 3 and 4 weeks (Fig. 4a).

At P 6, 8, and 10 weeks, the total number of type I TH-IR cells was significantly decreased and the number of type II TH-IR cells increased in strobe-reared retinas, but statistical significance was observed only at P 6 weeks compared to control retinas (Fig. 4b, c).

The total number of TH-IR cells showed a decreasing tendency for type I TH-IR cells and an increasing tendency for type II TH-IR cells in the strobe-reared retina (type I TH-IR cells decreased to 26.4 % and type II TH-IR cells increased to 54.0 % from P 3 to 10 weeks) (Fig. 4b, c).

### Functional assessment by ERG

**Fig. 5:**
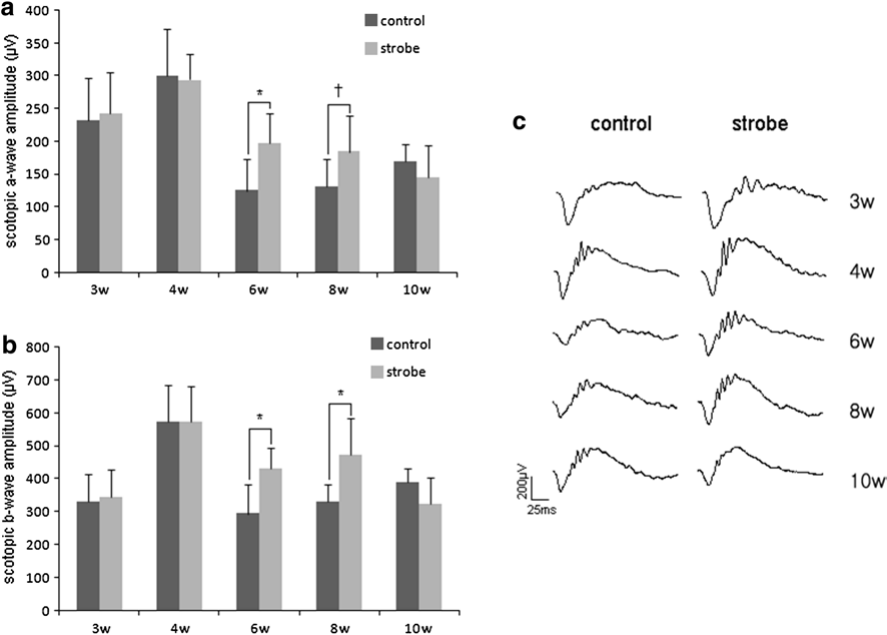
Electroretinogram (ERG) recordings taken from the eyes of control and strobe-reared rats (**a**, **b**). Representative examples of ERG responses are shown in order to compare the changes of a- and b-wave in control and experimental eyes. **c** Both a- and b-wave amplitudes increased at postnatal 6 (6w) and 8 (8w) weeks in strobe-reared rats. No significant difference was observed at postnatal 3 (3w), 4 (4w), and 10 (10w) weeks. (*p < 0.05, ^†^ *p* < 0.1)

ERG recordings were taken to evaluate the retinal function of strobe-reared rats. Both a- and b-wave amplitudes increased in strobe-reared rats at P 6 and 8 weeks (Fig. 5). Significant differences were not observed in a- and b-wave amplitudes at P 3 and 4 weeks. However, a-wave ERG responses increased by 58 % at P 6 weeks and 10 % at P 8 weeks, and b-wave ERG responses increased by 46 % at P 6 weeks and 43 % at P 8 weeks in strobe-reared rats compared to control rats (a-wave, 125.0 [49.0] and 131.3 [42.0] μV vs. 197.4 [44.9] and 184.4 [55.1] μV; b-wave, 294.3 [89.6] and 330.9 [53.7] μV vs. 430.0 [63.3] and 473.7 [111.3] μV at P 6 and 8 weeks, respectively). These differences disappeared at P 10 weeks (a-wave, 169.5 [26.2] μV vs. 145.0 [48.6] μV; b-wave, 388.2 [43.2] μV vs. 324.7 [77.5] μV).

## Discussion

Dopaminergic amacrine cells are a particularly well-described population of retinal neurons among the wide-field amacrine cells that receive input from the cone bipolar cells (Hokoc and Mariani 1987). Immunohistochemical techniques have determined that the rate-limiting enzyme for dopamine synthesis, TH, is localized to these cells (Nguyen-Legros et al. 1984; Mariani and Hokoc 1988; Dacey 1990; Mitrofanis and Provis 1990; Mills and Massey 1999). Dopaminergic retinal neurons depend on a light stimulus for complete development. This dependence was indicated by experiments in which rats were dark-reared throughout the period of dopaminergic cell development. These rats showed reduced staining intensity of dopaminergic cells (Kato et al. 1980) and reduced dopamine storage (Parkinson and Rando 1983).

Retinal dopamine is necessary for high-resolution, light-adapted vision (Jackson et al. 2012). Dopaminergic cells seem to interact with retinal circuits to suppress signal flow through rod circuits and enhance signal flow through cone circuits (Witkovsky 2004); thus, the contribution of dopamine to day-/night-induced alterations of retinal processing is believed to be important. Furthermore, dopamine plays multiple trophic roles in retinal function related to circadian rhythmicity, cell survival, and eye growth (Witkovsky 2004).

In this study, rearing under stroboscopic illumination throughout the period of development changed the function and morphology of TH-IR cells. TH-IR cells showed marked plastic changes in numbers and soma sizes. Type I TH-IR cells showed a decreased number and had larger somata after P 6 weeks in strobe-reared animals. Type II TH-IR cells showed an increased number after P 6 weeks in strobe-reared animals, but only a few regions showed statistical differences.

Dopamine is released during both light stimulation and total darkness in dopaminergic amacrine cells, and the relationship between dopamine release and light intensity is U-shaped (Li and Dowling 2000). Stroboscopic illumination that includes light stimuli with intense contrast and strength may influence dopamine release. According to Rohrer et al. (1995), form-deprivation myopia (FDM) in chickens appears to reduce the rate of dopamine synthesis in the light-adapted retina, and the normal rate of dopamine synthesis in the light can be restored by exposure to strobe light for 1 h. These authors stated that strobe light stimulates dopaminergic amacrine cell and increases TH activity in FDM chickens. Therefore, continuous stimulation by stroboscopic illumination during development presumably altered the morphology and the number of dopaminergic cells in this experiment. The changes in the ERG response also reflect a disturbance in the development of dopaminergic amacrine cells. Dopamine inhibits both scotopic a- and b-waves, and the dopaminergic blocker haloperidol greatly increases both scotopic a- and b-waves (Shulman and Fox 1996; Skrandies and Wässle 1988). Injection of the neurotoxin 6-hydroxydopamine (OHDA) causes degeneration of dopaminergic cells and additionally increases a- and b-wave amplitude (Naarendorp and Sieving 1991). The ERG response in this study is similar to that of a haloperidol or 6-OHDA model. We postulate that the increased a- and b-waves in this study resulted from abnormal dopamine release in the dopaminergic cells of the strobe-reared retina. These changes in the ERG response recovered at P 10 weeks. It appears that the morphological changes of the dopaminergic cells were maintained during the later periods of development, although this functional change could have been reversed via a compensatory mechanism between P 8 and 10 weeks. Many groups (Thornton et al. 1996; Pasternak 1987; Pasternak and Leinen 1986) have studied impairment of motion perception induced by strobe rearing, but the mechanism of this phenomenon is unknown. According to Mora-Ferrer and Gangluff (2000), dopamine influences movement detection through D2 dopamine receptors and the D2 dopamine receptor antagonist, sulpiride, and reduces sensitivity to the moving stimulus. In light of the ERG responses in this experiment, continuous exposure to a strobe light may act like a dopamine antagonist. Impaired motion perception in strobe-reared animals could be explained by abnormal dopaminergic amacrine cells that function like a dopaminergic antagonist.

In this study, the total number of type I and type II TH-IR cells was maintained during development in the control group retinas, whereas a tendency for the type I TH-IR cells to decrease and the type II TH-IR cells to increase was observed in strobe-reared retinas. This suggests that strobe light contributes to the differentiation of neurons to type II TH-IR cells rather than to type I TH-IR cells.

In our previous study (Shin et al. 2012), rearing guinea pigs under stroboscopic illumination induced thinning of the ONL and abnormal processes of rod bipolar cells. Because the light intensity (60-W incandescent lamp, 300 Lux) was not strong enough to induce light damage (Organisciak and Vaughan 2010) and no apoptotic cells were apparent, the thinning of the ONL was presumably caused by a mechanism other than light damage. Cheng et al. (2004) reported that rearing guinea pigs under strobe light induces myopia, and eyeball elongation is a potential explanation for ONL thinning. However, no significant ONL changes were observed in this rat model, which may have been because of species differences or experimental conditions. Responses to light stimuli can vary widely according to species or strain. Lavail et al. (1987) reported that the change in ONL thickness varies among different strains and species, even when the light exposure is the same. In light of these facts, the different response to strobe light may have been because of species difference. Guinea pig and rat retinas have many developmental and histological differences. For example, retinal development is precocial in the guinea pig, but is altricial in the rat (Spira 1975), and this developmental difference maybe one reason for the observed differences. Another putative explanation for the different response is variations in experimental conditions, such as the light intensity used in this study.

Further investigations are necessary regarding dopamine storage and release and TH phosphorylation in the strobe-reared animal model.
